# Bibliography

**Published:** 1891-02

**Authors:** 


### BIBLIOGRAPHY.

Dental Surgery: Including Special Anatomy and Pathology. By Henry Sewill, M.R.C.S., L.D.S. (Eng.). Third Edition, with 206 Illustrations. P. Blakiston, Son & Co., Philadelphia, 1890.

This well-known work of Dr. Sewill, now in its third edition, has many points of great value. It is a gratification, on opening a book, to be met at once with illustrations that exactly define the text. It is rare to meet anything better in this direction than is presented on page 11. Dentine is here represented as faithfully as it could be seen under the microscope. The same can be said of the chapter on "Development of Teeth," where the illustrations, taken directly from the specimens, leave no room for questioning or criticism. The old mode of drawings and woodcuts invariably bring in the personal equation and are necessarily subject to doubt and misapprehension.

These remarks apply with particular force to the exceedingly interesting and instructive chapter on "Caries." The fac-simile reproduction of photo-micrographs are here very fine, illustrating the action of micro-organisms in dentine, and confirm the recent observations of Miller, and the previous ones of Underwood and Milles. The whole chapter bears evidence of personal research and thorough work. The author gives credit to Professor Miller for having produced artificial caries; but evidently regards Underwood and Milles as the ones entitled to the largest consideration in connection with this subject. American readers, while willing to give due credit to the gentlemen named, cannot feel that they made a marked advance over the earlier work of Leber and Rottenstein.

The following quotation is hard to comprehend:

"Gallippe and Hoppe-Seyler, both have made some observations going to show that teeth (enamel and dentine) increase in density as age advances; but these observations are incomplete and fallacious, and it would be unsafe to base conclusions upon them. Since individual teeth of a set in the vast majority of cases vary considerably in structural character, it would be necessary in order to prove alteration in density to cut sections from the same tooth at different periods of its existence."

If the author means to be understood that there is no change in enamel, and especially in dentine, as age advances, he places himself in opposition to recognized clinical facts. If there is one thing settled, it is that function causes physiological action, and that dentine, at least, becomes, by increase of density, more resistant to caries as age advances. The operators in this country depend on this fact to enable them to keep the soft teeth until at a later age they can be filled with better material than ordinary plastics.

While on the subject of caries the author takes occasion to say, "That in face of all the facts and considerations, an American writer within late years has elaborately described what he styles inflammation of enamel and inflammation of dentine. . . . The publication of a statement of this kind seems hardly compatible with possession of an adequate knowledge of microscopical and bacteriological science."

The portion devoted to devitalization of pulp is open to criticism. It is surprising that an operator of the skill of the author should recommend wax as a cover-stopping for arsenic. It is wholly unreliable as a stopping in the most careful hands, and in those of students it is almost certain to result in failure, if not serious injury to surrounding parts.

It is further a matter of surprise that the author does not allude to the resistance offered by pulps, in a state of inflammation, to the action of arsenious acid. The treatment of this class of pathological presentations has been so much advanced in the past few years, and is so important, that the omission is a serious mistake.

In the treatment of pericementitis there is a very proper allusion to the necessity of care to render the canals aseptic; but that on the counter-irritation method, as applied to the gum, is of a routine character. It is about time some attention should be paid to the reasons why aconite and iodine should or should not be used. The topical action of aconite is, in the writer's estimation, if not injurious, at least of very little value. The effect of this agent is to paralyze the peripheral sensory nerves, and must, therefore, necessarily lessen the circulation at the part where an increase of the flow of blood is most to be desired.

The paragraphs devoted to erosion are equally defective, in view of the light thrown on this subject of recent years.

As a manual this work can be most heartily recommended. It has serious faults of omission, but these do not materially detract from its general value.

Descriptive Anatomy of the Human Teeth. By G. V. Black, M.D., D.D.S. Published by the Wilmington Dental Manufacturing Company, Philadelphia, 1890.

Dr. Black has shown in this volume, as he has in former productions of his pen, a very satisfactory attention to detail and correct description.

In the preface he says, "By my experience as a practitioner, as a teacher, and in my intercourse with fellow-practitioners, I have become convinced of a serious defect in the teaching of the details of the anatomy of the teeth and in the systematization of the terms used in their description." He further says, in the same preface, "The absence of a bibliography may be noted. The plan and object of this work has not seemed to call for many references to authorities. This does not imply, however, that authors who have preceded me, . . . to whom we are greatly indebted, have been either overlooked or ignored." This must be regarded as a serious defect in this and previous productions of this writer. It is a bad system and one that eventually robs the originator of an idea of all credit for work that may have cost him serious toil in investigation to bring out results. There could be no better plan devised than this to completely bury an original worker, and we are surprised that Dr. Black should persistently set an example that may react on his labors in the future.

The book itself will be invaluable to those who desire the anatomy of the human teeth in the minutest detail, both in the deciduous and the permanent series.

The part devoted to the consideration of "pulp-chambers" is novel in some of its illustrated features, in that it gives exact reproductions of these in silhouettes. His plan is original, and worthy of special notice. "After the pulp-chamber is exposed so that half of its concavity remains in the half of the tooth and has been ground smooth and flat, it should be inked on an ink-pad (such as is used for rubber stamps for printing) and a print made from it. This will give the form of the tooth and pulp-chamber."

This book can be most cordially recommended to the student and teacher as the clearest and most exact of any of the publications on the anatomy of the human teeth.

A Compend of Chemistry, Inorganic and Organic, including Urinary Analysis. By Henry Leffman, M.D., D.D.S., Professor of Chemistry in the Woman's Medical College of Pennsylvania, in the Pennsylvania College of Dental Surgery, and in the Wagner Free Institute of Science; Food Inspector for the Pennsylvania State Board of Agriculture. Third Edition revised. Pp. 193. Cloth, $1.00. Interleaved, for taking notes, $1.25. P. Blakiston, Son & Co., 1012 Walnut Street, Philadelphia, 1890.

The experience of Professor Leffman as a teacher is in itself a guarantee of the thorough adaptation of this work to the needs of the student, and the demand for a third edition is sufficient proof of the substantial aid which it affords in following a course of lectures on chemistry. As stated in the preface, "Any one who has had experience in teaching at American medical colleges knows that, as long as the present methods continue, some such assistance is absolutely essential. It affords to the student an opportunity to keep up with the lectures, and obviates the necessity of taking voluminous notes, in which serious errors are liable to occur. Such books, of course, cannot claim any originality; their merit lies in their accuracy, perspicuity, and judicious selection of facts."

These conditions have been admirably fulfilled in the work under consideration. The selection of facts, while full, is well chosen and compendious, while the perspicuity of their treatment is such as to almost warrant a claim for originality, in this phase of the work at least. While books of this character are not intended in any sense to replace the larger text-books, they will continue to find favor with the student as reliable aids in acquiring a knowledge of the subjects of which they treat. This one is a most satisfactory example of its class. E. C. K.

A Compend of Dental Pathology and Dental Medicine. By Geo. W. Warren, D.D.S., Clinical Chief of Pennsylvania College of Dental Surgery. Illustrated. P. Blakiston, Son & Co., Philadelphia, 1890.

This class of books is not as a rule to be commended, as it is impossible to condense satisfactorily the subject-matter to the limits desired. In the present instance, the author has endeavored to embrace Dental Pathology into the limits of forty-seven small pages, with unsatisfactory results to each of the subjects treated.

The balance of the book, fifty-nine pages, is devoted to "Dental Medicine." This is more successful, and doubtless will have a value to such as simply desire condensed paragraphs describing the various agents used.

The Latin Grammar of Pharmacy and Medicine. By D. H. Robinson, Ph.D. Professor of Latin Language and Literature, University of Kansas, with an Introduction by L. E. Sayre, Ph.G., Professor of Pharmacy, etc. P. Blakiston, Son & Co., Philadelphia, 1890.

It may seem strange to some that a work of this kind should be deemed necessary, and in the countries of the Old World, where professional men are invariably recruited from the scholarly classes, it would doubtless be regarded as a reflection, and in some sense it must so be considered here. A previous knowledge of Latin has not, as yet, been made a prerequisite for admission in all medical and dental schools, hence a large number—happily growing fewer yearly—enter college with no training in this language. They are, necessarily, placed at once in an unfortunate position. It is to meet this class that the author has designed this valuable book. In his preface he gives the reasons for its preparation. "It was designed expressly to meet the needs of the first year pharmacy and medical students of this institution. Considerable experience in teaching such students had clearly shown that those who had not studied Latin were at a great disadvantage, compared with those who had acquired a fair knowledge of that language."

As far as the writer is aware, no such book has been before attempted. Dr. Thomas, in his medical dictionary (appendix), endeavors to supply this long-felt want; but it has not the merit of a work devoted exclusively to the subject. The author, therefore, in striking out in a new field, has certainly met the necessities of the class for whom it is intended, and which may also prove a valuable book of reference for those more favored.

The arrangement of the book is practical, the student gradually becoming familiar in the exercises with medical and pharmaceutical terms.

Essentials of Anatomy and Manual of Practical Dissection, etc. By Charles B. Nancrede, M.D. Third Edition. With 30 pages lithographic plates in colors and 180 illustrations. 388 pages. W. B. Saunders, Philadelphia, 1890.

This really valuable work, by Dr. Nancrede, now in its third edition, is intended, as the author states in his original preface, "to embody only those facts which have appeared to him to be really the 'essentials of anatomy.'" That this has met a want among students, and is a convenient book of reference to the general practitioner, is evident from the large sales.

The present edition has been enriched by thirty full-page colored plates, taken from the works of Maclise, Savage, Nuhn, and Hirschfeld, which so beautifully illustrate the topographical features that, as Dr. Edw. Martin says in his preface, "the student can compare his dissections at a glance." Such plates have not only a value in this direction, but add greatly to the beauty of the volume, from an artistic stand-point.

The Medical Bulletin Visiting-List; or, Physician's Call Record. F. A. Davis, Philadelphia, 1891.

The Physician's All-Requisite Time and Labor-Saving Account-Book. Designed by W. A. Seibert, M.D. F. A. Davis, Philadelphia, 1891.

Both of these are issued for the benefit of physicians, and combine so much that is of practical value that they seem to cover all the necessities of the busy practitioner as far as the keeping of accounts. The latter book is complete in its arrangement and fully justifies its title,—"Time and Labor-Saving."

THE PHYSICIAN'S VISITING-LIST FOR 1891. P. Blakiston, Son & Co., Philadelphia.

This is too well known to need special commendation. The fact that it is in its fortieth year is sufficient evidence that it has become a necessity to the busy practitioner.

FRIESE'S DENTAL BOOK. H. D. Justi, Philadelphia, 1891.

This is one of the best of this class of memorandum-books. It is conveniently arranged with diagrams for marking. It has the almost fatal objection that they all have that the date and day of the week are left blank. This must be inserted by the operator, and few will take that trouble.

